# Huisgen 3 + n Dipolar Cycloaddition Reactions: An Accessible and Useful Tool in Modern Organic and Heterocycle Synthesis

**DOI:** 10.3390/molecules28155692

**Published:** 2023-07-27

**Authors:** Ionel I. Mangalagiu

**Affiliations:** Faculty of Chemistry, Alexandru Ioan Cuza University of Iasi, 11 Carol 1st Bvd, 700506 Iasi, Romania; ionelm@uaic.ro

Heterocycle derivatives have been reported as invaluable compounds in agriculture, medicine, pharmacy, opto-electronics, and other fields [1,2,3]. Huisgen [3 + n] cycloaddition reactions represent a valuable and efficient tool in the design of heterocycle structures, consisting of the addition of a 1,3-dipole to a multiple-bond dipolarophile. This Special Issue (SI) focuses on Huisgen 3 + n dipolar cycloaddition reactions as a useful and powerful tool in the synthesis and chemistry of new heterocycle derivatives. The SI provides an update of relevant and important original contributions [1,2,4,5,6,7,8] and reviews [3,9] pertaining to these reactions, which have brought about significant advances in the development of the field of heterocycle derivatives.

The first original contribution to the SI is focused on the zwitterionic intermediates in the [3 + 2] cycloaddition reactions between *C*-arylnitrones (1,3-dipol) and perfluoro 2-methylpent-2-ene as a dipolarophile [4]. As for its mechanisms, DFT calculations have revealed that the [3 + 2] cycloaddition reactions occur via a one-step mechanism, irrespective of the nature of the reactants.

Another original contribution to the SI pertains to the [3 + 2] cycloaddition reaction of aryl-substituted nitrile *N*-oxides with trichloronitropropene as a dipolarophile [5]. It was found through experimentation that these cycloaddition reactions follow a two-stage, one-step mechanism. The DFT calculations have confirmed the experimental setup, and indicated that the reactivity differences in the series of *N*-oxides derive from their different nucleophilic activations and the polar character of the reactions.

The next original contribution is related to the synthesis of substituted pyrazoles via a [3 + 2] dipolar cycloaddition reaction cycloaddition of sydnones with acetylenedicarboxylate (DMAD) as a dipolarophile [6]. The authors synthetise new polyhalogenated N-arylglycines, 3-arylsydnones, and 1-arylpyrazoles containing a fluorine atom within their molecules. The reactions of sydnones with DMAD occur as typical Huisgen [3 + 2] cycloaddition reactions. 

Another original contribution to the SI has focused on obtaining new synthetic members of the Cinchona alkaloid family containing triazole derivatives by using the [3 + 2] Huisgen cycloaddition of azide to dipolarophile with a triple bond [7]. The triazole moiety was synthetised from the corresponding azido-cinchonine (1,3-dipol) with trimethylsilylacetylene (dipolarophile). In this way, the authors have synthetised a library of derivatives of quincorine and quincoridine, compounds that have the quinuclidine ring of the Cinchona alkaloids at both ends. 

The next details the synthesis of hybrid conjugated triazolyl-substituted (oligo)phenothiazine organosilicon derivatives with luminescence and reversible redox characteristics [8]. The synthesis of conjugated triazolylsubstituted (oligo)phenothiazine moiety was performed via the [3 + 2] cycloaddition reaction of azide [(azidopropyl)tri-alkoxysilanes, 1,3-dipol) with ethynylated (oligo)phenothiazines (dipolarophile). The hybrid conjugated compounds are intensively luminescent and present reversible redox systems according to cyclic voltammetry. The authors indicate that these favorable electronic properties could be easily included in the mesoporous hybrid materials.

Another original contribution is about the synthesis and biological activity (antcancer) of a selection of new cyano-substituted pyrrolo- (iso)quinoline derivatives [1]. The synthesis of pyrrolo[1,2-a]quinoline and pyrrolo[2,1-a]isoquinoline derivatives was performed via the [3 + 2] cycloaddition reactions of (iso)quinolinium ylides (1,3-dipole) to dipolarophile with double-bonded fumaronitrile. The obtained compounds were tested for their anticancer activity against a panel of 60 human cancer cell lines, and some of the compounds were found to demonstrate excellent activity.

The last original contribution to the SI is about the synthesis of pyrrolophthalazine cycloadducts via conventional thermal heating (TH) and microwave (MW) and ultrasound (US) irradiation [2]. The synthesis of cycloadducts was performed via Huisgen [3 + 2] dipolar cycloaddition reactions of phthalazinium ylides (1,3-dipole) to dipolarophile with triple-bonded methyl propiolate (MP) and or DMAD. The authors have also achieved a thorough comparative study on the efficiency of synthesis under conventional TH versus MW and US irradiation. Synthesis via MW or US irradiation has certain advantages: higher yields, decreasing of the reaction time and consumed energy, and decreasing of the amount of solvent used; consequently, these reactions could be considered environmentally friendly.

Finally, this SI contains two extensive reviews related to the [3 + 2] cycloaddition reactions applied in the azaheterocycles series. One of these reviews discusses [3 + n] cycloaddition reactions applied in the pyridazine and phthalazine series and their applications in medicinal chemistry and optoelectronics [3]. The synthesis of pyridazine and phthalazine derivatives were achieved via Huisgen [3 + n] dipolar cycloaddition reactions of various ylides (1,3-dipole) to different dipolarophiles with double and triple bonds, symmetrically or non-symmetrically. The stereochemistry, regiochemistry, and chorochemistry of the cycloaddition reactions are discussed. Applications in optoelectronics (in particular, fluorescent materials and sensors) and medicinal chemistry (in particular, pertaing to antimicrobials and anticancer) are also reviewed.

The other reviews offer new insights on indolizines and their benzo-derivatives [9]. By using the cycloaddition reactions of acetylene dipolarophiles to indolizines, various cyclazines derivatives have been obtained. The reviews also offer interesting mechanistic insights related to adducts and cycloadducts, and the theoretical aspects (one- or two-step mechanisms) thereof.
